# The value of cardiopulmonary ultrasound in diagnosing cardiogenic pulmonary edema in patients with heart failure

**DOI:** 10.3389/fmed.2026.1938070

**Published:** 2026-10-05

**Authors:** Yanfen Zhong, Jie Shen, Jingtao Li, Hongyu Zheng

**Affiliations:** Department of Ultrasound, Guangxi Academy of Medical Sciences & The People’s Hospital of Guangxi Zhuang Autonomous Region, Nanning, Guangxi, China

**Keywords:** cardiogenic pulmonary edema, cardiopulmonary ultrasound, diagnosis, heart failure, patients

## Abstract

**Objective:**

Heart failure (HF) is generally complicated with cardiogenic pulmonary edema (CPE), the early diagnosis and treatment is essential. We aimed to evaluate the effects of cardiopulmonary ultrasound (CPUS) in the diagnosis of CPE in HF patients, to provide reference for the management of HF.

**Methods:**

HF patients entering The People's Hospital of Guangxi Zhuang Autonomous Region & Guangxi Academy of Medical Sciences intensive care unit (ICU) between March 2025 and July 2026 were included. All patients underwent CPUS and chest computed tomography (CT), and comparison of the detection rates of cardiogenic pulmonary edema between CPUS and CT.

**Results:**

A total of 117 patients with HF were enrolled in this study. Of the 117 HF patients, 46 were diagnosed with CPE, and 43 were correctly diagnosed by CT, with a diagnostic accuracy of 94.9%, the positive predictive value (PPV) was 93.5%, the negative predictive value (NPV) was 95.8%, the sensitivity was 93.5%, the specificity was 95.8%, the positive likelihood ratio (PLR) was 22, and the negative likelihood ratio (NLR) was 0.07. While 41 were correctly diagnosed by CPUS, with a diagnostic accuracy of 92.3%, the PPV was 91.1%, NPV was 93.1%, the sensitivity was 89.1%, the specificity was 94.3%, the PLR was 16, and the NLR was 0.12. Comparison of CT and CPUS in the diagnosis of CPE, there was no statistically significant difference between the two methods (*X*^2^ = 0.111, *P* > 0.05). And The Cohen's kappa for the diagnosis of CPE by CT and CPUS is 0.873 (95% CI was 0.782–0.963, *P <* 0.001).

**Conclusions:**

In conclusion, the present study suggests that CPUS is feasible for identifying cardiogenic pulmonary edema in ICU patients with heart failure. Based on diagnostic agreement analysis, CPUS demonstrates promising performance for CPE detection in this critically-ill cohort.

## Introduction

Heart failure (HF) is associated with high mortality rates and unsatisfactory long-term prognoses, currently ranking as the leading cause of death among individuals with cardiovascular disorders (1). As a critical and severe complication of HF, cardiogenic pulmonary edema (CPE) is a key predictive factor for adverse outcomes. It significantly increases patients' risks of emergency department attendance and hospital readmission, and is therefore regarded as a core therapeutic intervention target for HF management (2).

PE refers to the imbalance of the production and return of tissue fluid in the lung due to some reasons, so that a large amount of tissue fluid cannot be absorbed by the pulmonary lymph and pulmonary venous system in a short period of time. It leaks from the pulmonary capillaries and accumulates in the alveoli and lung interstitium, causing serious obstacles to lung ventilation and ventilation. Clinically, it is characterized by extreme dyspnea, sitting breathing, cyanosis, profuse sweating, paroxysmal cough accompanied by a large amount of white or pink foamy sputum, and the lungs are full of symmetrical wet rales. CPE progresses rapidly and has a high mortality rate (3). Therefore, early diagnosis and timely treatment of CPE are of great significance to improve the prognosis and quality of life of HF patients.

Pulmonary capillary wedge pressure (PCWP) measurement has long been regarded as the gold standard for the clinical diagnosis of CPE. Nonetheless, this invasive examination method is complicated to operate and entails high medical costs, which greatly restricts its widespread routine use in clinical practice. And CPE has long been mainly dependent on chest x-ray or CT examination (4). Although bedside chest x-ray is more convenient, it also has its limitations. In addition to radiation, it is also affected by organs and tissues in the shadow of the mediastinum, such as the esophagus, trachea, and lymph nodes et (5, 6). Chest CT radiation is larger, which limits repeated use. Therefore, we still need to find new inspection methods to improve the sensitivity and specificity of the diagnosis of CPE, and to determine the severity of the disease (7). CPUS, a newly developed examination method in recent years, offers great convenience for clinical diagnosis and disease management. Traditional single-modality cardiac ultrasound focuses primarily on cardiac structure, systolic and diastolic function, and valvular abnormalities, which can reflect the underlying cardiac dysfunction but cannot directly visualize real-time pulmonary congestion. In contrast, lung ultrasound enables direct visualization of extravascular lung water through B-line artifacts, providing sensitive and dynamic information on pulmonary hemodynamic overload that cannot be fully captured by cardiac ultrasound alone. CPUS can be used to evaluate key factors which affect patient's prognosis including the structure and function of the heart, pulmonary artery pressure and pulmonary edema, and these two modalities can be performed sequentially using the same machinery and probe (8). With the continuous deepening of clinical research, the value of bedside cardiopulmonary ultrasound in detecting different lung diseases and pleural lesions has been reassessed. It has advantages of feasible bedside, non-radioactive, reusable and low cost (9). Therefore, CPUS has emerged as a promising bedside strategy for improving the accuracy and timeliness of CPE identification and congestion monitoring in critically ill HF patients.

Over the past 5 years, lung ultrasound research has evolved substantially beyond conventional diagnostic accuracy studies. Contemporary investigations increasingly leverage lung ultrasound for prognostic stratification in acute and chronic heart failure, prediction of clinical decompensation, congestion-guided therapeutic management, serial outpatient monitoring, longitudinal follow-up of disease trajectory, and integration with established clinical risk scores. Notably, the clinical role of lung ultrasound has gradually evolved from a simple diagnostic tool for distinguishing pulmonary edema etiology to a practical monitoring method for serial assessment of congestion changes, enabling dynamic evaluation of disease progression and treatment response (10–12). These advances reflect a growing recognition that lung ultrasound provides not only a snapshot of pulmonary congestion but also dynamic and actionable information that can inform clinical decision-making across the continuum of care.

Despite these important developments, a fundamental and unresolved question persists: how does point-of-care CPUS perform relative to chest CT, the current reference standard for the anatomical assessment of pulmonary oedema? While prior studies have compared lung ultrasound with chest radiography or clinical assessment, head-to-head quantitative comparisons between CPUS and chest CT specifically for the diagnosis and grading of CPE remain limited, particularly in the HF patients.

Moreover, there were relatively few studies on the application of CPUS, most of which focus on the correlation between pulmonary ultrasound and echocardiography. In the study, we sought to investigate the value of CPUS for comprehensive and assessment of the degree of CPE in patients with heart failure. Thereby providing evidence for the management of HF patients.

## Methods

### Participants

We enrolled HF patients entering The People's Hospital of Guangxi Zhuang Autonomous Region & Guangxi Academy of Medical Sciences intensive care unit (ICU) and meeting the inclusion criteria between March 2025 and July 2026. Inclusion criteria: patients met the diagnostic criteria of heart failure (13). The definitive diagnosis of CPE served as the reference standard in the present study and was independently determined by a multidisciplinary adjudication team comprising two attending cardiologists and one senior respiratory physician. All panel members were fully blinded to the results of chest CT and CPUS examinations to avoid incorporation bias. The diagnostic criteria included: (1) the presence of acute or progressively aggravated respiratory distress or orthopnea, (2) bilateral pulmonary rales on physical auscultation, elevated jugular venous pressure, (3) elevated serum BNP or NT-proBNP levels, (4) obvious resolution of respiratory symptoms following standardized anti-heart failure therapy. Patients who fulfilled the comprehensive composite diagnostic criteria were enrolled as CPE-positive cases. All adjudicated diagnostic results were finalized prior to imaging data analysis and statistical calculation, and were used as the gold standard for evaluating the diagnostic efficacy of CT and CPUS. Exclusion Criteria involved patients with incomplete data, those referred to an external center, and individuals with chronic obstructive pulmonary disease or pulmonary thromboembolism.

Written informed consent was acquired from all participants, and the study protocol was approved by the Ethics Committee of the People's Hospital of Guangxi Zhuang Autonomous Region (KY-GZR-2025-104). This investigation was conducted in accordance with the Declaration of Helsinki.

### Data source

Demographic, laboratory, and clinical data were retrieved from electronic medical records, including: (1) Demographic information: the patient's admission number, gender, age, vital signs at admission, NYHA functional class, cardiovascular comorbidities (hypertension, diabetes mellitus, coronary artery disease), alcohol, smoking. (2) Laboratory data: N-terminal pro-B-type natriuretic peptide (NT-pro-BNP), Tropnin-I (Tn-I), creatinine (CREA), Cystatin-C (Cys-C). (3) Current heart failure medications: norepinephrine, dopamine, and recombinant human brain natriuretic peptide.

### Chest CT examination

The chest CT examination results of the patients were collected through the picture Archiving and Communication System (PACS). In the study, chest CT was employed as a practical reference standard for comparative analysis, rather than an infallible diagnostic criterion. The primary observation focuses on identifying manifestations of cardiogenic pulmonary edema in patients, including cardiomegaly, pericardial effusion, interlobular septal thickening, gravity-dependent ground-glass opacities, flocculent and patchy pulmonary shadows, pleural effusion, and Kerley B lines.

### Cardiopulmonary ultrasound examination

Transthoracic echocardiographic measurements were performed according to the 2015 American Association of Echocardiography (ASE) recommendation (14), using the commercially available Mindray M9 color Doppler ultrasound or Philips IE33 equipped with the S5-1 probe (frequency range of 1–5 MHz). All CPUS examinations were performed strictly following standardized bedside ultrasound protocols. System gain was consistently adjusted to ensure clear visualization of tissue structures and artifact patterns, with identical depth and time-gain compensation settings applied for all patients to guarantee image uniformity. All measurements were carried out by the experienced physician who was blinded to the objectives of the study to maintain an unbiased assessment. Image quality control was implemented throughout the study: only images with clear visualization of anatomical landmarks and satisfactory resolution were included for final analysis. An independent imaging score committee, composed of 2 blinded expert sonographers, reviewed all images. For intra-observer variability assessment, one experienced ultrasonographer repeated the offline image analysis two weeks apart, blinded to the initial results. For inter-observer agreement evaluation, two independent senior physicians performed blinded image interpretation.

Patients were placed in the supine or left lateral decubitus position. Left ventricular ejection fraction (LVEF) was measured using the Simpson method, which was adopted as a technical parameter to evaluate left ventricular function. Dynamic ultrasound images were stored, and relevant indicators were recorded including LVEF, ventricular dimensions, pulmonary artery systolic pressure, left atrial size.

Pulmonary ultrasonography was performed in accordance with the bedside pulmonary ultrasound in emergency (BLUE) protocol, which was summarized by Professor Lichtenstein in 2008 (15). A standard 8-zone image acquisition approach was used: the anterior area is from the anterior axillary line to the parasternal line, and the lateral region is from anterior axillary line to posterior axillary line. The anterior area was divided into anterior superior area and anterior inferior area, and the lateral area was divided into lateral superior area and lateral inferior area by the horizontal line equivalent to the second and third intercostal area and its extension. The observation focuses on A-lines, B-lines (the number of B-lines visualized in each view), pleural effusion, and lung consolidation. Dynamic images were stored, and all related parameters were recorded. The ultrasonic diagnostic criteria for pulmonary edema were defined as the presence of diffuse B-lines in unilateral or bilateral lungs; pulmonary edema was diagnosed when ≥8 B-lines were detected in a single region or the interval between adjacent B-lines was≤3 mm.

Combined with the results of cardiac and pulmonary ultrasonography, CPE was determined. The ultrasonic diagnostic criteria for cardiogenic pulmonary edema were defined as LVEF < 50% on echocardiography, disappearance of normal A-lines unilaterally or bilaterally on pulmonary ultrasound, and diffusely distributed B-lines (≥8 B-lines in one scanning area, or the interval between adjacent B-lines ≤ 3 mm). Some patients are accompanied by unilateral or bilateral pleural effusion.

All enrolled patients underwent both chest CT and CPUS examinations within a short time window. The interval between the two examinations was median 3.3 ± 1.5 h. During the inter-examination period, some patients received routine standardized clinical interventions for acute cardiogenic pulmonary edema, including diuretic administration, oxygen therapy adjustment, and non-invasive ventilatory support. All patients received continuous and standardized clinical management throughout the examination interval. Although individual patients received symptomatic treatment, the short examination time window avoided clinically significant dynamic changes in pulmonary edema severity, which ensured the comparability of imaging results between the two examinations.

### Statistical analysis

R software (version 4.0) was utilized for all statistical analyses. Categorical data were expressed as *n* (%), and the chi-square test was used for comparison of the detection rates of cardiogenic pulmonary edema between CPUS and CT. And *P* < 0.05 was considered statistically significant. Cohen's Kappa statistic was calculated to determine diagnoses consistency between CT and CPUS and inter-observer agreementand intra-class correlation coefficient (ICC) was used to assess intra-observer reproducibility.

## Results

### CT manifestations of cardiogenic pulmonary edema

The baseline characteristics of the study group are presented in Table 1. A total of 117 patients with heart failure were enrolled in this study. The main CT manifestations of CPE include cardiomegaly, pericardial effusion, interlobular septal thickening, gravity-dependent ground-glass opacities, flocculent and patchy pulmonary shadows, pleural effusion, and Kerley B lines (Figure 1). Of the 117 HF patients, 46 were diagnosed with CPE, and 43 were correctly diagnosed by CT (Table 2).

**Figure 1:**
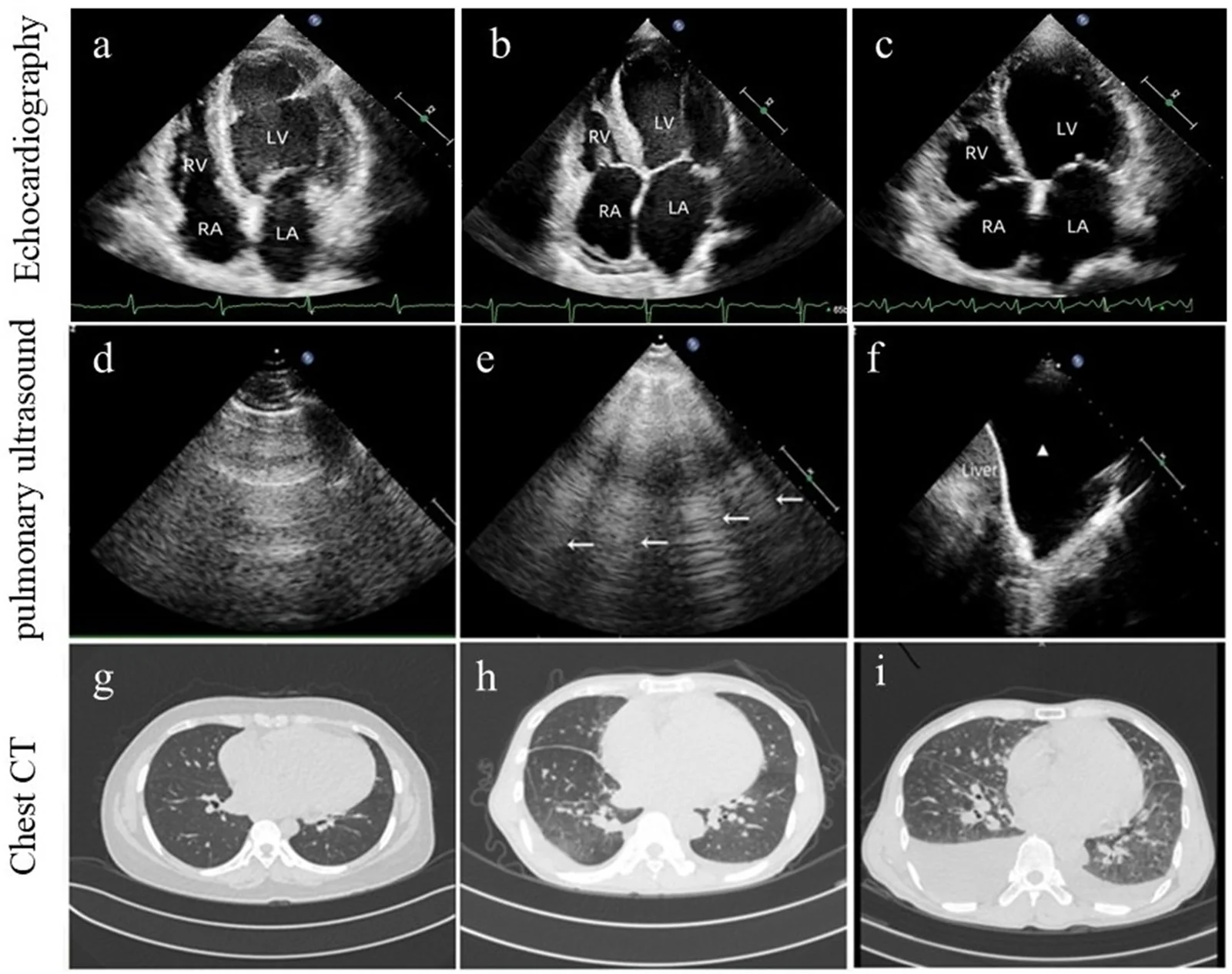
Comparison of cardiopulmonary ultrasound and chest CT diagnosis cardiogenic pulmonary edema. **(a–c)** Echocardiographic manifestations including cardiomegaly, pericardial effusion and cardiac function decreased; **(d–f)** pulmonary ultrasound manifestations including increased number of B lines (white arrow), pleural effusion (white triangle area); **(g–i)** chest CT mainly showed thickening of interlobular septa, flocculosity, patchy high-density image and pleural effusion with bronchial signs.

**Table 1:** Baseline characteristics of the patients.

Baseline characteristics	Result
Age (years)	59.78 ± 13.73
Gender	
Male (case/%)	78 (67%)
Female (case/%)	39 (33%)
Weight (kg)	71.23 ± 8.89
Cardiovascular comorbidities	
Hypertension (case/%)	81 (69%)
Coronary heart disease (case/%)	63 (54%)
Diabetes (case/%)	54 (46%)
History of alcohol and smoking	
Alcohol (case/%)	66 (56%)
Smoking (case/%)	58 (50%)
Vital signs	
Systolic BP (mmHg)	144.55 ± 14.66
Diastolic BP (mmHg)	82.84 ± 10.09
Pulse rate (/min)	83.55 ± 11.70
Respiratory rate (/min)	19.11 ± 2.52
NYHA functional class (case/%)	
Grade I	0 (0%)
Grade II	4 (3%)
Grade Ⅲ	49 (42%)
Grade Ⅳ	64 (55%)
Echo parameters	
LVEF (%)	34.82 ± 12.79
LVEDD (mm)	60.48 ± 7.76
LAD (mm)	47.07 ± 7.76
SPAP (mmHg)	51.96 ± 12.95
Laboratory data	
NT-Pro-BNP (pg/mL)	6,786.26 ± 652.84
Tn I (ng/mL)	1.12 ± 0.26
CREA (umol/L)	143.55 ± 86.98
Cys-C (mg/L)	1.68 ± 0.83
Current heart failure medications	
Norepinephrine (mg)	27.62 ± 4.66
Dopamine (mg)	923.08 ± 79.68
Recombinant human brain natriuretic peptide (mg)	2.30 ± 1.63

BP, blood pressure; LVEF, left ventricular ejection fraction; LVEDD, left ventricular end-diastolic diameter; LAD, left atrial diameter; SPAP, systolic pulmonary artery pressure; Pro-BNP, pro brain natriuretic peptide; Tn I, tropnin-I; CREA, creatinine; Cys-C, cystatin.

**Table 2:** CT manifestations of cardiogenic pulmonary edema.

CT manifestations	*n* (%)
Cardiomegaly	43 (93.5)
Pericardial effusion	36 (78.3)
Interlobular septal thickening	32 (69.6)
Gravity-dependent ground-glass opacities	38 (82.6)
Flocculent and patchy pulmonary shadows	35 (76.1)
Pleural effusion	26 (56.6)
Kerley B lines	40 (87.0)

### Cardiopulmonary ultrasound manifestations of cardiogenic pulmonary edema

The main CPUS manifestations of CPE include cardiomegaly, pericardial effusion and LVEF < 50% on echocardiography, disappearance of normal A-lines unilaterally or bilaterally on pulmonary ultrasound, and diffusely distributed B-lines (≥8 B-lines in one scanning area, or the interval between adjacent B-lines ≤ 3 mm). Some patients are accompanied by unilateral or bilateral pleural effusion (Figure 1). The ICC for interobserver agreement was 0.84 (95% CI: 0.65–1.0), indicating excellent reproducibility. The kappa value for PUS scoring was 0.79 (95% CI: 0.60–0.98), indicating substantial interobserver agreement. Of the 46 HF patients with CPE, 41 were correctly diagnosed by CPUS (Table 3).

**Table 3:** CPUS manifestations of cardiogenic pulmonary edema.

CPUS manifestations	*n* (%)
Cardiomegaly	43 (93.5)
Pericardial effusion	37 (80.4)
LVEF < 50%	42 (91.3)
Disappearance of normal A-lines unilaterally or bilaterally	37 (80.4)
Diffusely distributed B-lines	34 (73.9)
Unilateral or bilateral pleural effusion	21 (45.7)

CPUS, cardiopulmonary ultrasound; LVEF, left ventricular ejection fraction.

### Comparison of CT and CPUS in the diagnosis of cardiogenic pulmonary edema

Among the 46 patients with CPE complicated by heart failure, chest CT detected 43 cases, the positive predictive value (PPV) was 93.5%, the negative predictive value (NPV) was 95.8%, with a diagnostic accuracy of 94.9%, the sensitivity was 93.5%, the specificity was 95.8%, the positive likelihood ratio (PLR) was 22, and the negative likelihood ratio (NLR) was 0.07 (Table 4). CPUS identified 41 cases, the PPV was 91.1%, NPV was 93.1%, with a diagnostic accuracy of 92.3%, the sensitivity was 89.1%, the specificity was 94.3%, the PLR was 16, and the NLR was 0.12 (Table 5). The Cohen's kappa for the diagnosis of CPE by CT and CPUS is 0.873 (95% CI was 0.782–0.963, *P <* 0.001). There was no statistically significant difference in the detection rates between the two methods (*X*^2^ = 0.111, *P* > 0.05, Table 6).

**Table 4:** Clinical diagnosis and CT diagnosis of cardiogenic pulmonary edema.

Clinical	CT		Sum
	Positive	Negative	
Positive	43	3	46
Negative	3	68	71
Sum	46	71	117

**Table 5:** Clinical diagnosis and CPUS diagnosis of cardiogenic pulmonary edema.

Clinical	CPUS		Sum
	Positive	Negative	
Positive	41	5	46
Negative	4	67	71
Sum	45	72	117

CPUS, cardiopulmonary ultrasound.

**Table 6:** Comparison of CT and CPUS in the diagnosis of cardiogenic pulmonary edema.

CPUS	CT		Sum
	Positive	Negative	
Positive	41	4	45
Negative	5	67	72
Sum	46	71	117

CPUS, cardiopulmonary ultrasound.

## Discussion

HF represents a major clinical critical syndrome induced by various pathogenic factors and organic cardiovascular lesions, with CPE serving as a frequent concomitant complication of this condition (16). In HF patients, the elevation of pulmonary capillary wedge pressure can trigger ultrastructural injuries to pulmonary capillary walls. Such pathological changes further result in the accumulation of extravascular lung fluid, which eventually progresses to the development of CPE. Characterized by rapid disease progression and extremely high mortality, CPE poses a severe threat to patient prognosis (17, 36). Accordingly, timely and accurate early diagnosis of CPE is essential for improving the survival outcomes of affected patients.

CPUS for the diagnosis of CPE was first applied to critically ill patients in the intensive care unit (ICU) by Lichtenstein et al. (18) for the evaluation of respiratory diseases and circulatory failure. The BLUE protocol and FALLS protocol proposed by him have become the diagnostic criteria for point-of-care CPUS. In a 218 patient multi centre cohort, Riishede et al. reported Focused cardiopulmonary ultrasound added to standard clinical examination in patients with signs of respiratory failure had no impact on the diagnostic accuracy, but significantly increased the proportion of appropriate treatment prescribed and the proportion of patients who spent less than 1 day in hospital (19). Moreover, it has advantages of feasible bedside, non-radioactive, reusable and low cost. In our study, among the 46 patients with CPE, the diagnostic accuracy of CPUS was found no significant difference with CT for CPE, and good consistency was observed. CPUS can serve as a reliable auxiliary examination method for this condition. This result is concordant with the previous study by Zhang et al. (20).

A systematic review and meta-analysis by Mhanna et al. (21), pooling studies, concluded that A point-of-care pulmonary ultrasound (PUS) is a useful tool to detect subclinical pulmonary edema, and outpatient PUS-guided diuretic therapy of pulmonary congestion reduces urgent visits for worsening symptoms of HF. whereas a further review by Chew et al. (22) emphasised the primacy of echocardiographic filling-pressure estimates. Our results add to, and in part reconcile, conflicting evidence summarised in prior systematic reviews which preserves the high sensitivity of B-lines while adding echocardiographic parameter LVEF specificity addresses this known trade-off and supports the pooled conclusion of Santus et al. (23) that no single parameter is sufficient. LVEF < 50% combined with diffuse bilateral B-lines on pulmonary ultrasound represents the characteristic CPUS findings of CPE. As a critical parameter for the clinical evaluation of global cardiac function, LVEF measured by echocardiography enables reliable assessment of cardiac systolic and diastolic performance and serves as a routine biomarker for echocardiographic diagnosis of heart failure. Decompensated HF is frequently complicated by varying degrees of pulmonary congestion and pulmonary edema. The onset of pulmonary edema reduces the lung air-to-fluid ratio. Aside from non-specific pulmonary manifestations such as pleural effusion and pulmonary consolidation, transthoracic lung ultrasonography typically detects pathognomonic hyperechoic bands with comet-tail artifacts, defined as B-lines.

Previous studies (2, 24) have found that HF patients with CPE have lower LVEF, more severe diastolic dysfunction, larger left and right ventricles, and higher pulmonary artery systolic pressure (SPAP). When the patient has left ventricular systolic and/or diastolic dysfunction, the left ventricular end diastolic pressure and left atrial pressure increase accordingly, which cause the left heart to increase, and the pulmonary venous pressure and pulmonary capillary hydrostatic pressure increase accordingly. EVLW is composed of intracellular fluid, alveolar fluid and pulmonary interstitial fluid. It is a fluid distributed outside the pulmonary blood vessels (25). In general, the intracellular fluid is relatively fixed, and the alveolar fluid and interstitial fluid can change, reflecting the degree of CPE. The B-line in the lung ultrasound signs is a reverberation artifact, derived from the edema thickened subpleural lobular septum and the alveoli with increased water content (26–28). The change in the number of B-line can reflect the change of EVLW. So CPUS can be used to diagnose CPE and monitor the effect of HF treatment, which is consistent with our findings.

Studies have shown that the number of B-lines varies with the amount of lung water. These changes reflect the severity of PE in a certain sense (29). A study evaluated the left heart function of patients admitted to the cardiovascular department through the application of echocardiography and pulmonary ultrasound, the results have showed that the number of B-lines is related to the patients' heart function classification and left ventricular radiography, and LVEF is closely related to the degree of diastolic insufficiency (30). Besides, it's been reported that pulmonary ultrasound examination can have a better predictive effect on the right heart pressure and PVR of patients with HF (31). In addition, more and more studies (32, 33) have shown that the index value that can clearly represent the decompensation of heart function in patients with HF is closely related to the B-line. Several studies (34, 35) have found that lung ultrasound can detect PE and accurately detect changes in the amount of fluid in lung tissue before the early clinical symptoms of HF patients appear. These results are consistent with our findings. It is worth noting that clear distinction should be drawn between ICU patients enrolled in the study and outpatient heart-failure cohorts. CPUS was applied here for diagnostic discrimination of CPE during critical illness. Outpatient cohorts typically comprise clinically stable chronic heart-failure individuals, in whom lung ultrasound serves mainly for prognostic stratification, identification of subclinical congestion prior to clinical decompensation, and longitudinal monitoring to adjust ambulatory decongestive treatment. Given these marked differences in patient physiology, disease phase, and clinical objectives, diagnostic thresholds and conclusions obtained from this ICU population cannot be directly extrapolated to outpatient, general wards or primary-care settings.

This study has certain limitations. Firstly, this study is a single-center study, and the number of cases included in this study was small. Due to the limited sample size, we did not perform subgroup stratification or sensitivity analysis to quantitatively evaluate the impact of inter-examination time intervals and intervening treatments on diagnostic performance. Secondly, due to the poor specificity of the B-line, it can only indicate the presence of pulmonary interstitial syndrome, but it cannot clearly show that the specific causes of PE. It is necessary to combine specific etiology and medical history, patient clinical manifestations, and echocardiography in clinical application. Thirdly, the investigation is fundamentally a diagnostic accuracy study comparing CPUS with chest CT for the identification of pulmonary oedema, and it does not directly address the rapidly expanding frontiers of lung ultrasound research, including prognostic stratification, prediction of decompensation, congestion-guided therapy, outpatient monitoring, longitudinal follow-up, or integration with clinical risk scores. Additionally, in the study, chest CT was employed as a practical reference standard for comparative analysis, Chest CT depicts parenchymal anatomical alterations suggestive of CPE, but it cannot directly quantify physiological haemodynamic status. Its interpretation may be confounded by concurrent pulmonary pathologies such as pneumonia, ARDS, and chronic interstitial lung changes.

## Conclusions

In conclusion, the study suggests that CPUS is feasible for identifying cardiogenic pulmonary edema in ICU patients with heart failure. Based on diagnostic agreement analysis, CPUS demonstrates promising performance for CPE detection in this critically-ill cohort.

## Data Availability

The raw data supporting the conclusions of this article will be made available by the authors, without undue reservation.
